# Cross-Linked Carboxymethyl Cellulose and Silk Proteins in Corneal Re-Epithelialization: A Case Series

**DOI:** 10.3390/jcm14186600

**Published:** 2025-09-19

**Authors:** Francesco Boselli, Fabio Scarinci, Romina Fasciani

**Affiliations:** 1 Ophthalmology, ASST Brianza, Vimercate Hospital, Via Santi Cosma e Damiano 10, 20871 Vimercate, Italy; francescoboselli@outlook.it; 2 Ophthalmology, Fondazione Policlinico Universitatio Agostino Gemelli IRCCS, Università Cattolica del Sacro Cuore, Largo Agostino Gemelli 8, 00168 Rome, Italy; romina.fasciani@gmail.com; 3 Ophthalmology Unit, San Giovanni-Addolorata Hospital, Via Santo Stefano Rotondo 6, 00184 Rome, Italy

**Keywords:** cornea, eye, wound healing, silk, CMC

## Abstract

**Background/Objectives:** Corneal re-epithelialization is a critical process following surgical procedures such as photorefractive keratectomy (PRK), phototherapeutic keratectomy (PTK), and corneal UV cross-linking (CXL), as well as cases of corneal abrasion. Delayed epithelial healing can lead to increased discomfort, a higher risk of infection, and suboptimal visual outcomes. This retrospective case series aims to evaluate the efficacy of a novel ophthalmic solution containing cross-linked carboxymethyl cellulose (CX-CMC) and silk proteins in promoting corneal re-epithelialization and improving post-surgical recovery. **Patients and methods:** A total of 15 patients who underwent PRK, PTK, or CXL or who presented with corneal abrasions were included in the study. Along with standard post-surgical treatment, patients received CX-CMC and silk protein-based eye drops (CORDEV, Ophtagon, Rome, Italy) six times a day. Corneal epithelial thickness was assessed using topography at follow-up visits. **Results:** Corneal re-epithelialization was observed in all subjects within 24 to 48 h post-procedure. The mean corneal epithelial thickness at 48 h was 73.21 µm, which falls within the typical range of a proliferating corneal epithelium. **Conclusions**: The CX-CMC and silk protein-based formulation accelerated corneal healing, achieving rapid epithelial recovery. This novel ophthalmic solution offers a promising alternative to conventional post-surgical treatments, potentially improving patient outcomes by reducing healing time, minimising discomfort, and lowering the risk of complications associated with delayed re-epithelialization.

## 1. Introduction

The cornea is a transparent connective tissue essential for vision, providing two-thirds of the eye’s refractive properties. It has five distinct layers: (1) the epithelial layer, which provides a protective barrier; (2) Bowman’s layer, a thin acellular zone conferring structural support; (3) the corneal stroma, which constitutes about 90% of the corneal thickness and contains organised collagen fibrils and keratocytes; (4) Descemet’s membrane, a dense basement membrane for the endothelium; and (5) the endothelial layer, a monolayer of hexagonal cells responsible for maintaining corneal transparency through active fluid regulation [1].

Of these layers, the epithelial cells are the most exposed to environmental damage, making corneal wound healing a significant medical concern [2]. This is particularly important given the high incidence of traumatic damage to the cornea and the increasing number of refractive surgeries performed. Corneal surgical procedures such as corneal cross-linking (CXL), photorefractive keratectomy (PRK), and phototherapeutic keratectomy (PTK) are widely used to treat corneal disorders, but they are associated with potential complications that may impact visual outcomes and patient recovery [3]. For example, one of the most common complications following CXL is delayed epithelial healing, which can lead to prolonged discomfort, corneal haze, and an increased risk of infection [4,5]. PRK, which involves epithelial removal to reshape the cornea, is also associated with delayed re-epithelialization, corneal haze, stromal remodelling abnormalities, and the risk of postoperative infection, particularly in cases of inadequate post-operative care [6,7]. Additionally, prolonged epithelial healing in PRK may contribute to suboptimal refractive outcomes and persistent dry eye symptoms [8]. Similarly, PTK, which is performed to remove superficial corneal opacities or irregularities, carries a risk of epithelial defects, stromal haze, and recurrence of the original pathology, particularly in cases of recurrent corneal erosion syndrome [9,10]. These complications highlight the importance of optimising post-operative healing strategies to reduce epithelial healing time, minimise inflammation, and lower the risk of vision-threatening complications.

Re-epithelialization involves the migration, proliferation, and differentiation of corneal epithelial cells to cover the defect created by the surgery. The healing process and time can vary depending on the surgical technique, the extent of epithelial removal, and patient-specific factors such as age, existing comorbidities, and post-operative care [9,11]. Corneal re-epithelialization following PRK typically occurs within 3–7 days [6,8,12]. Similarly, following CXL, studies have documented re-epithelialization occurring within 3 to 40 days post-CXL [4,5,13], where most eyes reach a quiescent state; however, several complications can arise during this period. Since delayed or impaired re-epithelialization can significantly impact patient outcomes, there is growing interest in therapies such as eye drops that could enhance and expedite this process. Indeed, eye drops that promote corneal re-epithelialization are crucial for post-operative care after refractive surgeries or CXL, as they foster healing and reduce the risk of complications [14]. Faster healing associated with better visual outcomes can lead to improved patient satisfaction and a reduction in post-operative complications. Studies have shown that prompt re-epithelialization not only minimises the risk of infection and corneal complications but also contributes to a more stable refractive outcome, reducing the likelihood of corneal haze and scarring, which can impair long-term vision quality [7].

Ongoing advancements in this field are leading to the creation of new formulations that focus on both the speed and quality of re-epithelialization. Tailored eye drops with innovative formulations have the potential to greatly enhance the cornea’s natural healing process. This could result in faster recovery times and provide an effective method for post-operative care. Ultimately, such advancements could reduce discomfort, improve quality of life, and help prevent long-term complications.

In line with that, this study provides a description of corneal re-epithelialization in post-PRK, PTK, and CXL patients and patients with corneal abrasion, using an eye drop containing cross-linked carboxymethyl cellulose and silk proteins (CXC-SP). These novel components have shown promise in accelerating wound healing, further underscoring the importance of innovative treatments in post-surgical management.

## 2. Materials and Methods

### 2.1. Patient Population

The present case series reports the data derived from 15 subjects who underwent surgical procedures involving de-epithelialization or suffered from corneal abrasion. The subjects were of both sexes and ranged in age from 16 to 57. Data were collected from patients who were seen during routine clinical examinations as part of standard clinical practice. Eligible patients were those who underwent surgical procedures, including CXL, PRK, and PTK, as well as those with a first diagnosis of corneal abrasion who were not receiving any ongoing ophthalmic therapy at the time of diagnosis. Exclusion criteria included patients with known allergies or hypersensitivity to any components of the tested formulations or standard therapies, or those requiring different treatments compared to the rest of the study population. All patients provided written informed consent, the study adhered to the Declaration of Helsinki, and ethical approval was granted by the “Fondazione Policlinico Universitario A. Gemelli IRCCS” Hospital Ethics Committee, Rome (approval number: 7381, date: 30 April 2025).

### 2.2. Surgical Procedures

CXL: Under topical anaesthesia, the central 9 mm of the corneal epithelium was manually removed using a swab or a golf club sponge. An isotonic riboflavin 0.1% eye drop without dextrose (Vibex^®^ Rapid Avedro Inc., Waltham, MA, USA) was applied every 2 min for 10 min. Subsequently, UV-A irradiation was applied for 12 min with an accelerated pulsed protocol, delivering a total energy of 7.20 J/cm^2^ (Avedro KXL, 15 mW/cm^2^, 2 s on, 1 s off, USA).

PRK: Under topical anaesthesia, the central 8 mm of the corneal epithelium was manually removed using a swab or a golf club sponge. PRK was performed using topography-guided excimer laser ablation (Teneo, Bausch & Lomb, Bridgewater, NJ, USA). Each laser pulse removes approximately 0.25 microns of corneal tissue. Based on preoperative evaluations, a customised ablation plan was created.

PTK: Under topical anaesthesia, the central 8 mm of the corneal epithelium was manually removed using a swab or a golf club sponge. PTK was performed using an excimer laser (Teneo, Bausch & Lomb, Bridgewater, NJ, USA). Each laser pulse removed approximately 0.25 microns of corneal tissue. A masking agent, such as 0.7–2% hydroxypropyl methylcellulose (HPMC, Op’Cover, BVI Medical, Waltham, MA, USA), was applied to smooth the cornea during ablation. The procedure was adjusted based on several factors, including lesion depth, corneal pachymetry, and intraoperative surface regularisation.

### 2.3. Treatment

Patients received standard therapy following the surgical procedure, which includes netilmicin eye drops four times a day and ketorolac eye drops three times a day, alongside cyclopentolate eye drops three times a day. Moreover, silk protein and CX-CMC-based eye drops (CORDEV, Ophtagon, Rome, Italy) were instilled six times daily. Instead of the TCL, an occlusive bandage was preferred in these patients.

Systemic non-steroidal anti-inflammatory drugs (NSAIDs) were administered for analgesia in cases of pain. Patients were told to follow the therapy and return for a follow-up visit 48 h after treatment and 1 month after treatment.

### 2.4. Tomography

To evaluate and follow the re-epithelialization process, a slip lamp evaluation with fluorescine dye and tomography (MS39, CSO, Rome, Italy) measuring epithelium thickness were used.

### 2.5. Data Analysis

Data analysis was conducted using descriptive statistics. Continuous variables, such as corneal epithelial thickness, were summarised using the mean and standard deviation. Given the observational and non-comparative nature of this case series, no inferential statistical tests were performed. Descriptive statistics were performed using GraphPad Prism 10.2.2 version (GraphPad Software, San Diego, CA, USA).

## 3. Results

A total of 15 eyes from 15 patients were included: 40% were female and 60% were male. Mean Age was 28.53 years (age range 15–57 years). At 48 h post-procedure, epithelial thickness demonstrated consistent re-epithelialization patterns across corneal cross-linking (CXL), photorefractive keratectomy (PRK), phototherapeutic keratectomy (PTK), and corneal abrasion. In particular, the overall mean epithelial thickness was 73.3 ± 8.4 µm (range: 54.0–86.4 µm) (Table 1).

**Figure 1:**
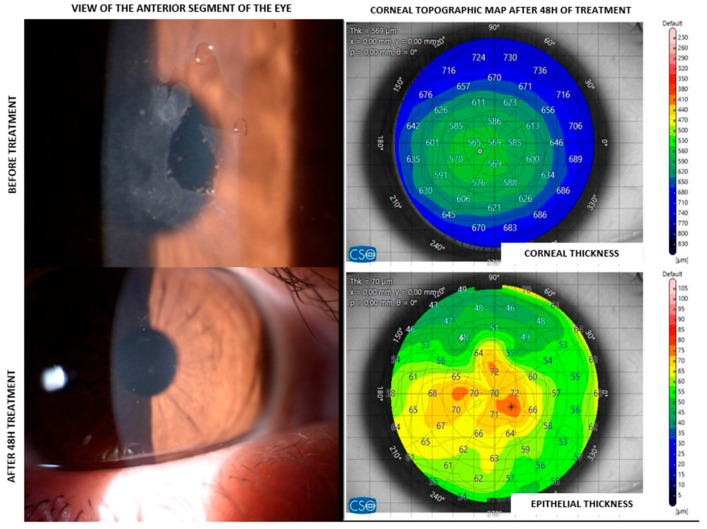
Clinical and topographical assessment of corneal healing in a CXC-SP patient with corneal abrasion. The left images depict the clinical appearance of the cornea before (**top**) and after (**bottom**) 48 h of treatment, showcasing the healing progression. The right images provide corresponding corneal (**top** map) and epithelial (**bottom** map) thickness maps in micrometres (µm).

Figure 1 shows one representative case of corneal abrasion, confirming that CXC-SP treatment promotes early re-epithelialization within 48 h, as demonstrated by the anterior segment images showing corneal surface restoration (left) and by the corneal maps (right), where the pachymetric and epithelial thickness distributions indicate epithelial coverage over the defect area.

At 48 h, mean keratometry values (Kavg) ranged between 38.28 D and 49.79 D across the sample. Corneal astigmatism (Cyl) varied widely, with values between –1.10 D and –4.72 D, distributed along different axes. The maximum keratometry (Kmax) spanned from 45.94 D (7.35 mm) to 66.44 D (5.08 mm), with the steepest profiles observed in patients treated with CXL and PRK. The minimum corneal thickness (ThkMIN) showed substantial variability, ranging from 405 µm to 589 µm, with thinner corneas predominantly observed in CXL and PRK cases (Table 2).

To monitor corneal re-epithelialization after CXC-SP treatment, regional epithelial thickness was evaluated at preoperative stage, at 48 h, and 1 month after treatment in 6 patients of the original cohort (2 PRK cases and 4 CXL cases) (Table 3).

At 48 h, ΔET (center vs. periphery) is consistent with an early repithelization process in which peripheral epithelial hyperplasia precedes central closure. By 1 month, ΔET values suggest that central epithelial thickening had occurred and that the corneal surface was approaching complete normalization.

## 4. Discussion

This retrospective study investigated corneal re-epithelialization following CXC-SP treatment in different clinical scenarios, including corneal abrasion, PRK, PTK, and CXL. Despite the heterogeneity of surgical indications, epithelial thickness measurements at 48 h consistently indicated early re-epithelialization, with values clustering around 73 µm. The representative case of corneal abrasion (Figure 1) confirmed these findings both clinically and topographically, supporting the hypothesis that CXC-SP promotes rapid wound coverage. Keratometry values at 48 h showed wide inter-patient variability. Despite this variability, early epithelial recovery was not delayed, reinforcing the potential of CXC-SP. Regional epithelial thickness changes (ΔET, centre vs. periphery) were assessed in the six patients who completed the 1-month follow-up. At 48 h, ΔET values are consistent with the early phases of epithelial healing. In this stage, epithelial cells migrate and proliferate more rapidly from the wound margins, leading to relative peripheral hyperplasia [15]. This behaviour is in line with the physiological mechanism of corneal repair. At 1-month follow-up, ΔET values suggest that the central epithelium had closed the defect. This pattern is compatible with the remodelling phase of corneal wound healing, in which the central epithelium tends to temporarily increase in thickness before reaching long-term stability [2]. Importantly, no adverse events such as stromal haze, infection, or persistent pain were detected at follow-up visit, confirming both the safety and tolerability of the treatment in the acute postoperative phase.

Corneal epithelial healing typically occurs within 3–4 days following CXL or PRK, while complete re-epithelialization generally takes at least 7–10 days post-surgery [13,16,17]. Various strategies have been explored to accelerate this process, with common approaches including therapeutic contact lenses (TCLs), anti-inflammatory drugs, and artificial tears [18]. Among these, TCLs have become a cornerstone of postoperative care, providing mechanical protection for the regenerating epithelium, reducing discomfort, and stabilising the ocular surface—factors that collectively create a more favourable healing environment [19]. However, despite their widespread use and benefits, therapeutic lenses may sometimes delay the healing process. Prolonged lens wear can interfere with the natural migration of epithelial cells, potentially slowing wound closure. Additionally, issues such as hypoxia, lens-induced mechanical friction, or suboptimal lens fit can further compromise the speed of epithelial regeneration [19].

Among eye drops, cross-linked hyaluronic acid has emerged as a promising component, significantly expediting healing with epithelial closure [13,20]. Further innovations in cross-linked hyaluronic acid formulations have reduced healing times for the majority of patients, achieving complete re-epithelialization by day 3 post-PRK [21]. Other promising agents, such as matrix therapy containing regenerative compounds, have shown similar efficacy in reducing healing times to around 2.7–3 days post-CXL or penetrating keratoplasty [17,22]. Trehalose combined with sodium hyaluronate has also demonstrated efficacy in shortening healing time to an average of 2.3 days after CXL, compared to 3.8 days with sodium hyaluronate alone [20]. However, all these formulations were consistently used in conjunction with TCL.

In this context, the present study introduces a novel formulation combining silk proteins and CX-CMC, which successfully achieves a rapid corneal re-epithelialization within 48 h without TCL. Therefore, in our study, corneal re-epithelialization appears faster than the time reported for standard regimens in the literature, even when advanced formulations such as cross-linked hyaluronic acid gels or matrix therapy agents are employed, which typically reduce healing time to approximately 2.7–3 days [17,22]. While the absence of a comparator arm prevents a direct demonstration of superiority, the observation that all patients achieved epithelial recovery within 48 h suggests that CX-CMC and silk protein eye drops may offer an advantage over conventional strategies.

Both silk proteins and CMC have been extensively studied for their wound-healing properties. CMC is known to enhance corneal epithelial cell attachment, migration, and re-epithelialization [23]. In a pioneering systematic review [24], evidence about CMC efficacy was inferred from 64 different clinical trials. The study supports the effectiveness of CMC in improving the signs and symptoms of dry eyes, pointing to CMC as an adjuvant treatment for various conditions, including anterior eye trauma, infection, inflammation, and discomfort associated with contact lens use. Additionally, the study emphasised that artificial tears containing CMC can enhance epithelial healing; this is attributed to the presence of hydrogel-like components, which are known to activate the epidermal growth factor (EGF) receptor. This activation promotes the healing of corneal epithelial wounds [25].At the same time, silk-derived proteins have demonstrated their capacity to accelerate epithelial migration, proliferation, and adhesion in both in vitro and in vivo studies [26,27]. Recently, silk proteins have also been proven to be effective in dry eye disease in a large clinical trial [28], demonstrating significant improvements in tear film stability, ocular surface integrity, and subjective symptom scores compared to standard lubricants. In this context, the present findings highlight the therapeutic potential of silk-derived biomolecules not only as lubricants but also as active promoters of ocular surface healing. Their biocompatibility, low immunogenicity, and ability to modulate cellular behaviour [29] make them particularly well-suited for use in post-surgical settings, where rapid and safe epithelial recovery is critical.

**Figure 2:**
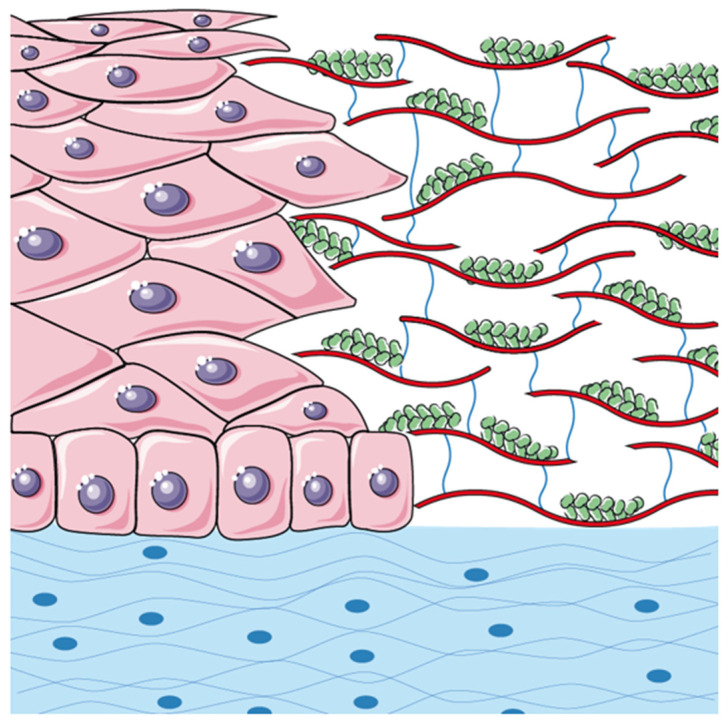
Silk-enhanced scaffold (SES) graphical representation. CX-CMC marked in red and blue; silk proteins marked in green. CX-CMC provides a structural scaffold that facilitates epithelial cell adhesion and migration, while silk proteins enhance cellular proliferation and differentiation, mimicking the natural extracellular matrix.

Interestingly, a study by Kundu et al. [30] provides compelling evidence for the regenerative potential of silk fibroin (a subunit of the silk fibre) and sodium carboxymethyl cellulose (NaCMC) blends. The research demonstrated that fibroin-NaCMC blends exhibit increased surface roughness, which enhances the films’ ability to create an optimised microenvironment that supports the cell’s adhesion, proliferation, and migration. Additionally, these films display beneficial swelling behaviour that improves moisture retention, making them effective for managing wound exudates and maintaining a moist healing environment. The improved mechanical properties of the films also make them more flexible and less brittle, allowing them to conform to wounds without breaking [30]. The cytocompatibility and wound healing capabilities demonstrated by these blended films underscore their potential as effective scaffolds for promoting epithelial healing, aligning with our findings on the accelerated wound closure observed with the CXC-SP solution. Building upon this foundation, we assume that the combined action of CX-CMC and silk protein significantly enhances the wound-healing capabilities of the ophthalmic solution, leading to the formation of a silk-enhanced scaffold (SES) (see Figure 2).

Specifically, CX-CMC provides a structural scaffold that facilitates epithelial cell adhesion and migration, while silk proteins enhance cellular proliferation and differentiation, mimicking the natural extracellular matrix [23,26,29]. This synergistic interaction is likely to accelerate epithelial recovery by promoting a more organised and stable epithelial layer, ultimately reducing healing time and minimising post-surgical complications [27]. Moreover, the hydrophilic and viscoelastic properties of CX-CMC contribute to the maintenance of a moist ocular surface, which is essential for epithelial regeneration and protection against external stressors [23]. Concurrently, silk proteins may exert additional bioactive effects, such as modulating inflammatory responses and reducing oxidative stress, further optimising the wound healing process [26,29].

Despite the encouraging results described, this study presents several limitations that should be acknowledged. First, the relatively small sample size and the retrospective, non-comparative design inevitably reduce the generalizability of the findings. The limited pool of subjects in preoperative and follow-up phases further constrained the longitudinal analysis, restricting the evaluation of regional values to only six patients. Moreover, the lack of a control group treated with conventional approaches, such as therapeutic contact lenses or artificial tears, makes it difficult to establish the precise contribution of CX-CMC and silk proteins beyond the natural course of corneal healing. Finally, the variability of the dataset should be taken into account. In particular, ΔET values showed substantial inter-patient variability, reflecting both the heterogeneity of clinical indications and the underlying structural alterations of keratoconic eyes.

Notwithstanding these limitations, the observation of consistently rapid re-epithelialization across all cases (Table 1) suggests that the proposed formulation could represent a valuable adjunct in postoperative management. By accelerating epithelial closure, CX-CMC and silk protein eye drops may reduce patient discomfort and pain, minimise the risk of infection, and lower the incidence of complications such as stromal haze. Faster epithelial recovery may also improve the stability of refractive outcomes, reduce the need for postoperative medications, and potentially obviate therapeutic contact lenses, which themselves may delay healing or increase infection risk. These advantages could translate into improved patient satisfaction, more efficient clinical management, and a lower burden of postoperative complications.

## 5. Conclusions

In conclusion, the present study offers an innovative strategy to achieve rapid corneal re-epithelialization. The rapid corneal healing observed and probably due to the formation of a silk-enhanced scaffold, represents a notable improvement over traditional recovery time. The safety and tolerability of this formulation, underscore its potential as a valuable component in post-operative eye care. The silk-enhanced scaffold could be a promising advancement in accelerating corneal healing after surgical procedures such as PRK and CXL, with the potential to improve visual outcomes, reduce infection risk, and minimise long-term complications.

## Figures and Tables

**Table 1:** Epithelial thickness measurements at 48 h post-procedure using CXC-SP.

ID	Age	Sex	Clinical History	Epithelial Thickness at 48 h (µm)
#001	30	F	Corneal abrasion	67.0
#002	29	M	CXL	74.2
#003	16	M	CXL	77.0
#004	29	F	CXL	62.8
#005	26	M	CXL	68.4
#006	25	F	PRK	75.8
#007	19	M	CXL	86.4
#008	35	F	PTK	54.0
#009	57	M	PTK	66.6
#010	32	M	PRK	73.6
#011	28	M	CXL	74.0
#012	46	M	PRK	82.3
#013	25	F	PRK	68.4
#014	15	M	CXL	76.1
#015	16	F	Corneal abrasion	82.9

**Table 2:** Keratometry and corneal values (48 h) post-surgery abrasion in patients treated with CXC-SP.

Patient ID	Kavg (D)	Cyl (D @ Axis)	Kmax (D/mm)	ThkMIN (µm)
#001	44.22	−1.49 D @ 43°	48.28 D (6.99 mm)	589
#002	44.49	−1.91 D @ 178°	48.53 D (6.95 mm)	510
#003	45.55	−2.59 D @ 12°	50.85 D (6.64 mm)	534
#004	43.40	−3.82 D @ 11°	51.45 D (6.56 mm)	434
#005	46.26	−2.42 D @ 16°	60.74 D (5.56 mm)	446
#006	38.40	−1.23 D @ 177°	47.52 D (7.10 mm)	407
#007	43.24	−4.16 D @ 101°	58.52 D (5.77 mm)	530
#008	39.71	−1.10 D @ 2°	45.94 D (7.35 mm)	433
#009	38.28	−1.13 D @ 174°	47.10 D (7.16 mm)	489
#010	38.64	−1.21 D @ 23°	46.89 D (7.20 mm)	500
#011	49.79	−4.72 D @ 164°	66.44 D (5.08 mm)	405
#012	43.75	−2.74 D @ 9°	52.11 D (6.49 mm)	445
#013	40.26	−1.24 D @ 176°	46.72 D (7.21 mm)	498
#014	46.36	−3.31 D @ 15°	59.15 D (5.71 mm)	421
#015	42.47	−1.29 D @ 25°	49.03 D (6.88 mm)	512

**Table 3:** Mean epithelial thickness changes.

	Preoperative	48 h	1-Month
ΔET	0.7 ± 6.4 µm	−1.7 ± 6.5 µm	3.2 ± 7.5 µm

## Data Availability

The datasets presented in this article are not readily available due to privacy reasons. The data supporting the conclusions of this article will be made available by the authors upon request.
